# Successful management of multiple permanent pacemaker complications – infection, 13 year old silent lead perforation and exteriorisation following failed percutaneous extraction, superior vena cava obstruction, tricuspid valve endocarditis, pulmonary embolism and prosthetic tricuspid valve thrombosis

**DOI:** 10.1186/1749-8090-4-12

**Published:** 2009-02-24

**Authors:** Pankaj Kaul, Krishna Adluri, Kalyana Javangula, Wasir Baig

**Affiliations:** 1 Yorkshire Heart Centre, Leeds General Infirmary, Great George Street, Leeds, LS1 3EX, UK

## Abstract

A 59 year old man underwent mechanical tricuspid valve replacement and removal of pacemaker generator along with 4 pacemaker leads for pacemaker endocarditis and superior vena cava obstruction after an earlier percutaneous extraction had to be abandoned, 13 years ago, due to cardiac arrest, accompanied by silent, unsuspected right atrial perforation and exteriorisation of lead. Postoperative course was complicated by tricuspid valve thrombosis and secondary pulmonary embolism requiring TPA thrombolysis which was instantly successful. A review of literature of pacemaker endocarditis and tricuspid thrombosis along with the relevant management strategies is presented. We believe this case report is unusual on account of non operative management of right atrial lead perforation following an unsuccessful attempt at percutaneous removal of right sided infected pacemaker leads and the incidental discovery of the perforated lead 13 years later at sternotomy, presentation of pacemaker endocarditis with a massive load of vegetations along the entire pacemaker lead tract in superior vena cava, right atrial endocardium, tricuspid valve and right ventricular endocardium, leading to a functional and structural SVC obstruction, requirement of an unusually large dose of warfarin postoperatively occasioned, in all probability, by antibiotic drug interactions, presentation of tricuspid prosthetic valve thrombosis uniquely as vasovagal syncope and isolated hypoxia and near instantaneous resolution of tricuspid prosthetic valve thrombosis with Alteplase thrombolysis.

## Case presentation

**Figure 1:**
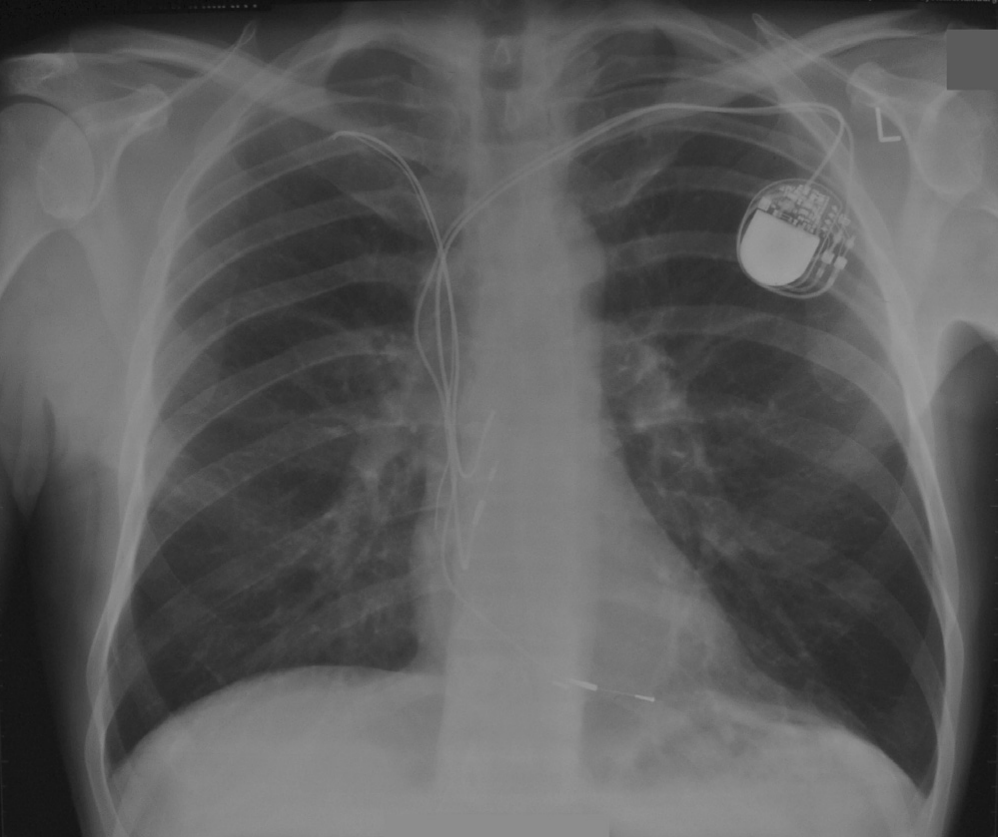
**Chest X ray (PA) showing 2 atrial and 2 ventricular pacing leads and 1 pulse generator in left infraclavicular pocket, the right sided pulse generator having been removed previously**.

**Figure 2:**
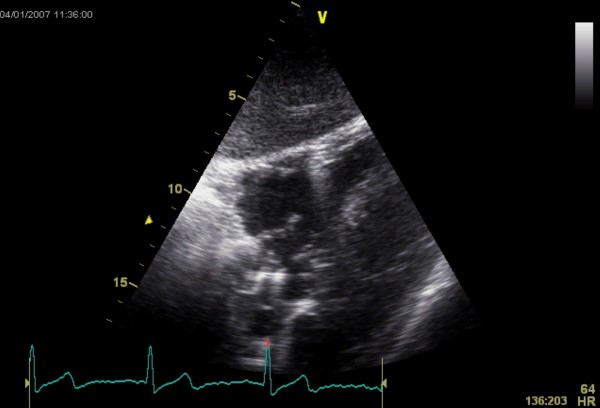
**Transthoracic echocardiogram showing a large right atrial vegetation**.

**Figure 3:**
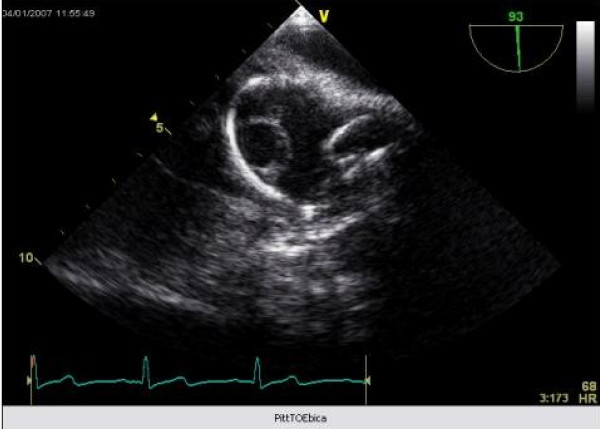
**Transthoracic echocardiogram showing vegetations in relation to the endocardial pacing lead**.

**Figure 4:**
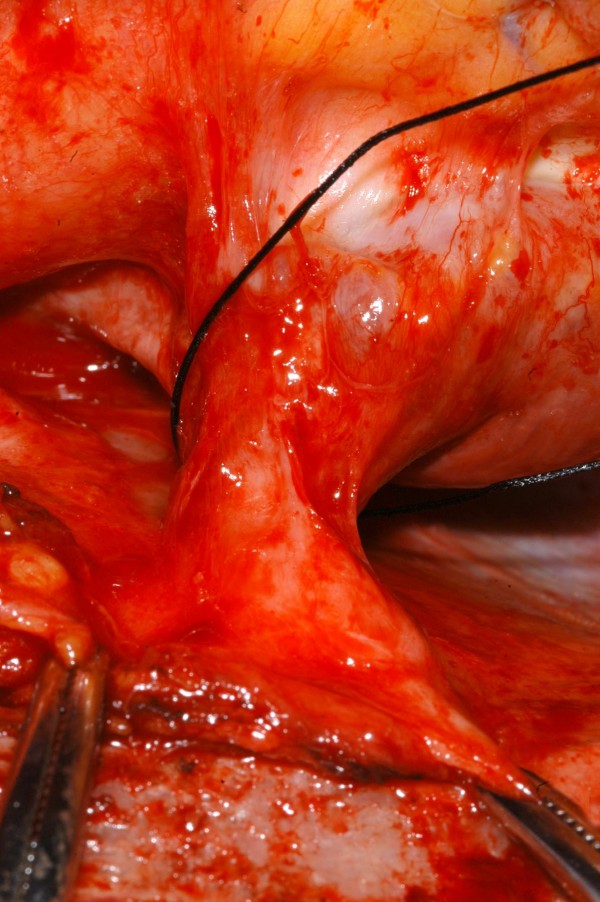
**Intraoperative photograph showing the tip of a pacemaker lead lying outside the right atrium, surrounded by inflammatory granuloma and densely adherent to the undersurface of the sternum**.

**Figure 5:**
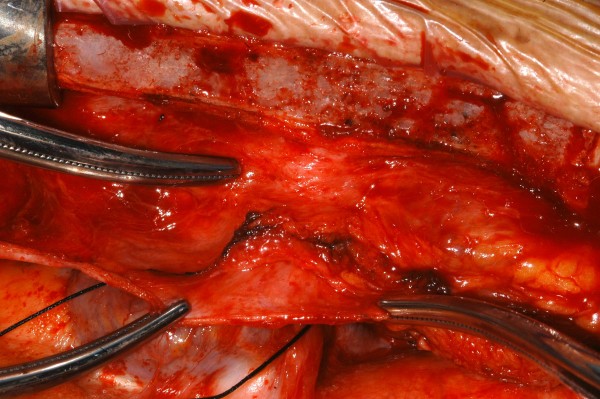
**Intraoperative photograph showing the extra pericardial location of the lead**.

**Figure 6:**
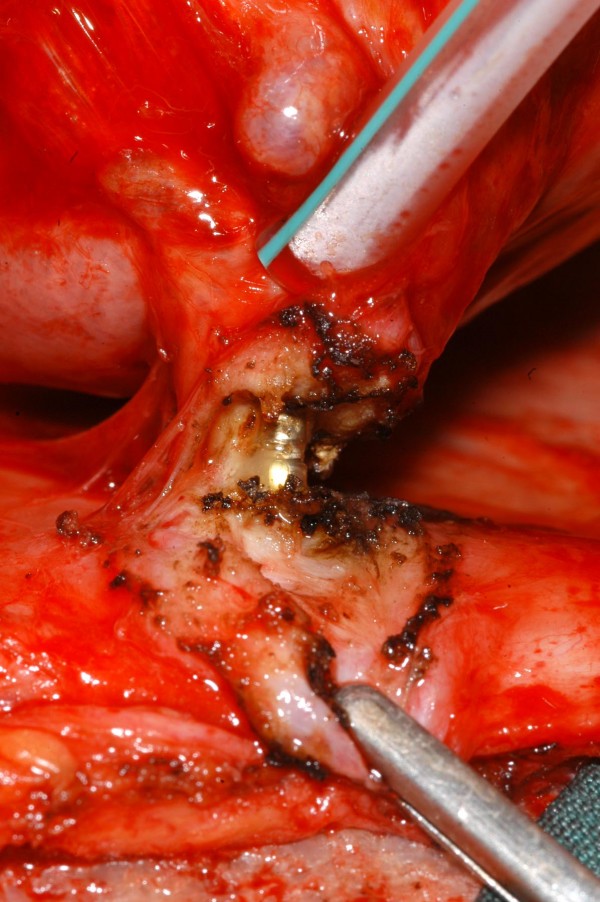
**Intraoperative photograph showing the inflammatory granuloma having been dissected to reveal the tip of the pacemaker lead**.

**Figure 7:**
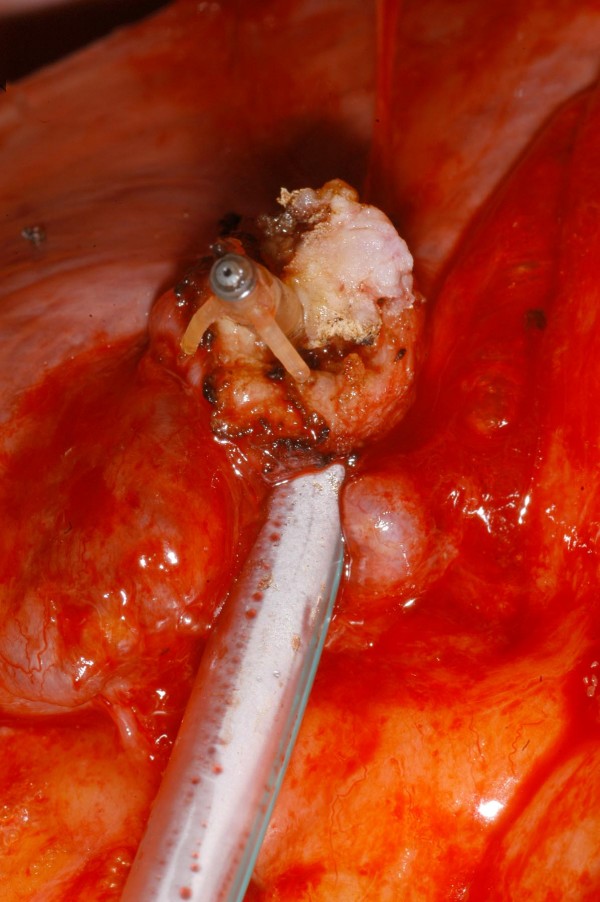
**Intraoperative photograph showing the tip of the pacemaker lead in greater detail**.

**Figure 8:**
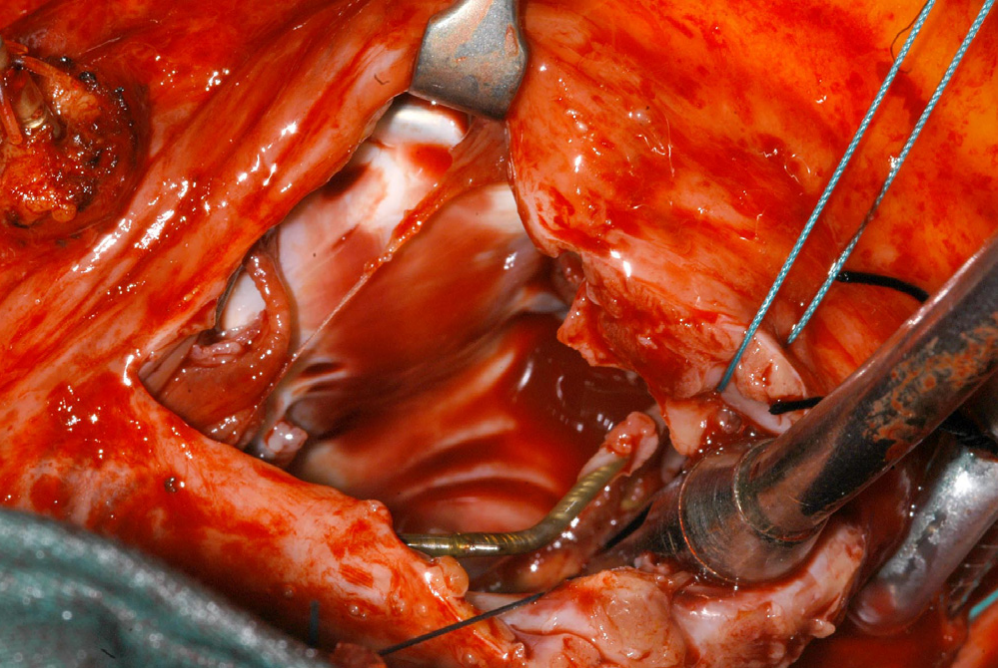
**Intraoperative photograph showing the right ventricular pacemaker lead surrounded by vegetations**.

A 40 year old man underwent dual chamber right infraclavicular pacemaker implantation for malignant vasovagal syndrome in 1988. This was complicated by two further reexplorations, one for a slipped atrial lead, and the second for primary wound dehiscence which required a pulse generator change. This was followed, in 1993, by infection of the pacemaker pocket by Staphylococcus epidermidis requiring pacemaker removal. However, only the pulse generator could be removed, and the percutaneous removal of the leads by traction had to be abandoned on account of cardiac arrest, in all probability, and with the benefit of hindsight, due to perforation of right atrium by the atrial lead during attempted percutaneous removal which consequently had to be abandoned. The patient was resuscitated successfully without taking recourse to median sternotomy, the leads were cut short and buried under pectoralis major and the wound closed. A fresh dual chamber pacemaker was implanted in left infraclavicular pocket, followed by an elective pulse generator change in 2001. Patient next presented in December 2006 with history of fever, rigors, night sweats and weight loss. Examination revealed bilateral pacemaker pocket infection and discharge and unremarkable cardiovascular examination. Chest X ray confirmed four pacing leads in the SVC, with two in right atrium and two in right ventricle, a pulse generator in the left infraclavicular pocket only (fig 1). Routine blood examination revealed white cell count of 20.3, neutrophils 17.3 and CRP 59. Bacterial culture of the wound discharge grew coagulase negative staphylococcus. Intravenous vancomycin and gentamycin was started. Vancomycin was changed to intravenous flucloxacillin after two days and oral rifampicin added subsequently. Transthoracic echocardiogram demonstrated large vegetations associated with the pacing leads at the atrial level, part of which prolapsed through the tricuspid valve during diastole (fig 2). Transesophageal echocardiogram revealed extensive and complex vegetation surrounding all leads in SVC, RA, RV and also involving tricuspid valve and right atrial and right ventricular endocardium (fig 3). At median sternotomy, one of the atrial leads was densely adherent to the under surface of the right half of the sternum just below the manubriosternal joint. It was surrounded by a large fibrous granuloma from the point it exited from the atrium till the point of its adherence to the under surface of the sternum (figs 4, 5). Presumably, the lead perforated the right atrium during the attempted percutaneous removal of the right sided pacing leads in 1993 which had to be abandoned when patient had had an asystolic arrest. Pericardial cavity was obliterated by adhesions. Cardiopulmonary bypass was established with ascending aortic and direct SVC and IVC cannulations. Caval tapes were snugged, temperature was allowed to only gently drift and right atriotomy was made on the beating heart. Both infraclavicular pacemaker pockets were exposed and the pulse generator on the left side removed after disconnecting the leads. The perforated atrial lead lying under the sternum, encased in the pericardial granuloma, was dissected out within the granuloma, excised at the level of the appendage and removed (figs 6, 7). The remaining portion of the lead, along with the other atrial lead, were removed after dissecting them out from the atrial endocardium along with the adherent thrombotic vegetations. There were large vegetations on the posterior and septal leaflets tricuspid valve leaflets and the chordae of the two leaflets had twined themselves around the ventricular pacing leads (fig 8). The endocardium of the inlet of the right ventricle was infiltrated by vegetations as well. The ventricular pacing leads were dissected off from the ventricular muscle and the tricuspid valve leaflets and chordae and removed. The tricuspid valve leaflets were excised completely, the adjoining atrial and ventricular infiltrated endocardium debrided and a 31 mm St Jude mechanical prosthesis stitched in place using 14 pledgetted interrupted 2 O ticron sutures. Atriotomy closed in a single layer. An epicardial pacemaker (Medtronic) was sutured in place and the pulse generator kept in a preperitoneal and retrorectus position in left hypochondrium and set at a rate of 90/min. Post procedure TOE revealed satisfactory prosthetic valve function and no residual vegetations in SVC, RA or RV. Patient received intravenous Flucloxacillin and oral rifampicin for a total duration of 6 weeks. Post operative period was uncomplicated apart from the requirement for large doses of warfarin to maintain INR in the required range, possibly as a result of antibiotic interactions. The postoperative transthoracic echocardiogram showed satisfactory tricuspid prosthetic valve function and patient was discharged home, 5 weeks after his operation.

**Figure 9:**
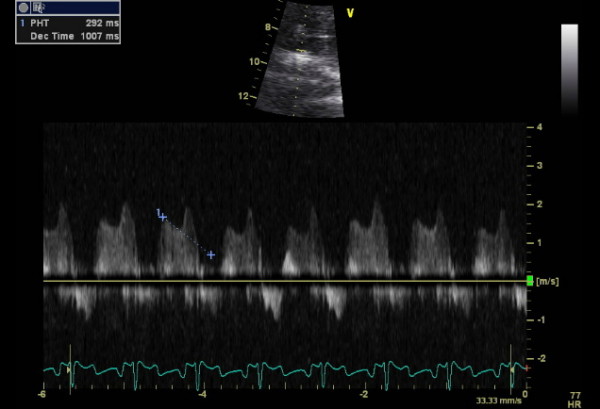
**Doppler echocardiogram showing a prolonged pressure half time of 270 msec suggestive of severe prosthetic tricuspid obstruction**.

**Figure 10:**
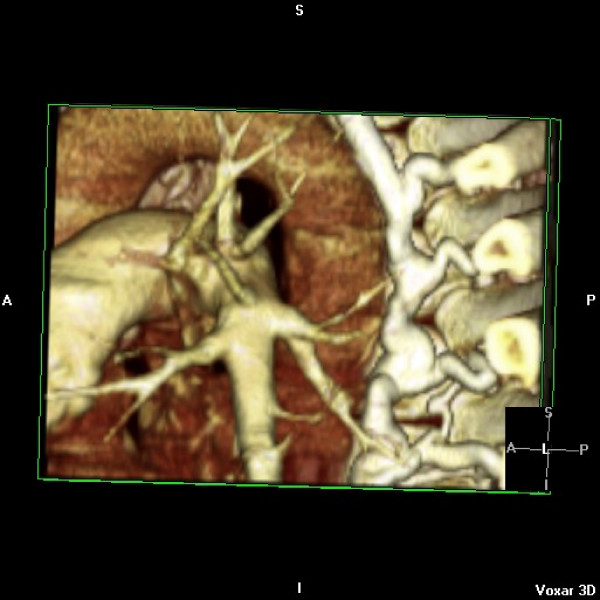
**Contrast CT angiogram of left pulmonary artery showing a "wispy" ill defined embolus in the upper lobar artery**.

**Figure 11:**
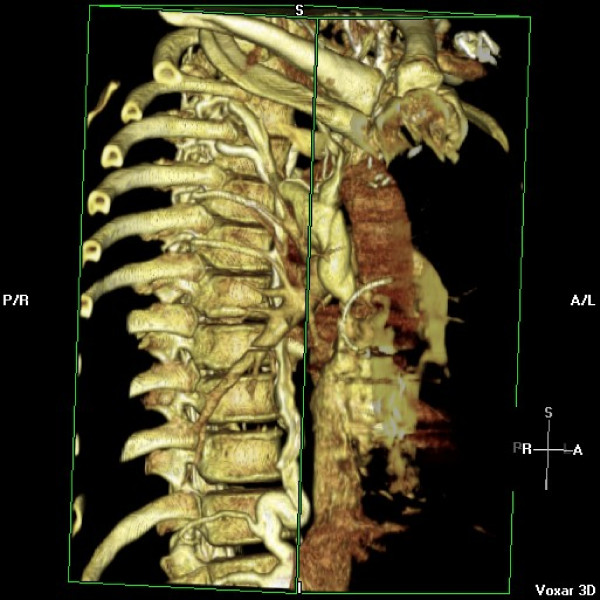
**Contrast CT angiogram showing extensive backfilling of azygous system from superior vena cava**.

**Figure 12:**
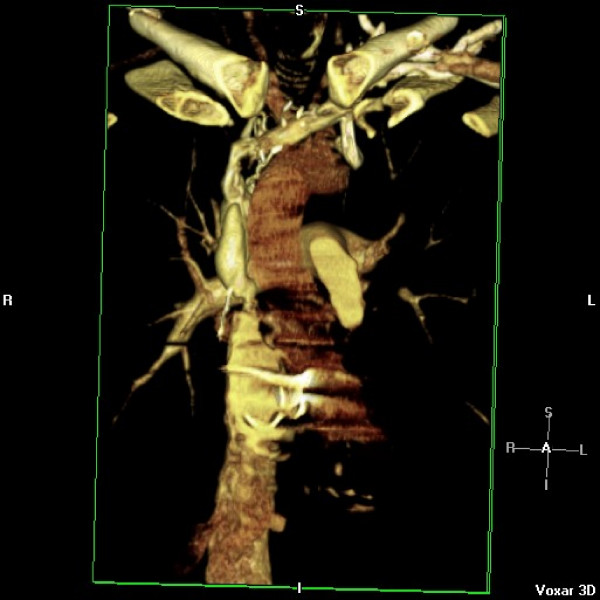
**Contrast CT angiogram showing residual stenosis both at the confluence of the two innominate veins and at the junction of SVC with right atrium**.

**Figure 13:**
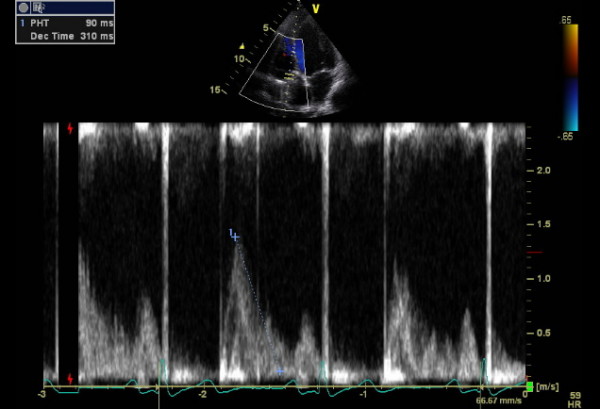
**Post thrombolysis Doppler echocardiogram showing complete resolution of tricuspid prosthetic thrombus as suggested by a normal pressure half time**.

He represented two weeks later with syncope while blood was being withdrawn for INR estimation. Examination revealed profound hypoxia with 88% oxygen saturations, no audible metallic heart sounds and 2 cm hepatomegaly. Arterial blood gas revealed pH 7.52, pO_2 _5.69, pCO_2 _3.64, HCO_3 _24.7. INR was suboptimal at 1.9. Transthoracic echocardiogram revealed antegrade flow across tricuspid prosthesis increased at 2.5 m/sec, prolonged pressure half time of 270 msec (fig 9) and an estimated effective tricuspid prosthetic orifice area of 0.8 cm^2^. No obvious valve occluder excursion, in contrast to the immediate postoperative echo, was seen and a small amount of mobile friable material was visualised on the ventricular side of the valve). Tricuspid valve fluoroscopy confirmed restricted occluder excursion. The right atrium was dilated with bulging of interatrial septum. Both ventricles were contracting well. A contrast CT angiogram of pulmonary arteries demonstrated a "wispy", ill defined embolus in left pulmonary artery extending into upper lobar artery which could have been chronic (fig 10). There was also evidence of extensive backfilling of the azygous system from the superior vena cava (fig 11), with some degree of residual stenosis both at the confluence of innominate veins and also at the junction of SVC with right atrium (fig 12). A diagnosis of tricuspid prosthetic thrombosis was made. Whether there was recent or old embolisation into PA was difficult to establish with certainty. Since the patient was haemodynamically stable and since his oxygenation improved somewhat with increasing the inspired oxygen concentration, it was elected to, in the first instant, to treat with aggressive anticoagulation with intravenous heparin and oral warfarin, aiming to keep both INR and APTT between 3.5 to 4.5. One week later, however, TOE continued to show significant prosthetic thrombotic obstruction with no clinical improvement. Patient was, therefore, thrombolysed with100 mgs of Alteplase given as a standard 3 hr infusion. Patient felt symptomatic improvement instantaneously, with oxygen saturations improving to 97% and later 100%, restoration of normal pulmonary gaseous exchange. Transthoracic echo showed no gradient across the tricuspid prosthesis, with normal pressure half time (fig 13). Fluoroscopy confirmed restoration of normal prosthetic leaflet excursions. Patient was discharged home, 2 weeks after his readmission, requiring 12 mgs of warfarin to keep his INR between 3.5 and 4.5. Patient is being followed up regularly in outpatient clinic and continues in NYHA class1 and has resumed all previous activities.

## Discussion

Pacemaker system infections can be generator pocket or lead infections and complicate 0.5 – 2 percent permanent pacemaker implantations [1]. The usual predisposing causes of infection include haematoma, erosion, long duration of procedure, operator's inexperience and reexploration [2]. Early infections are most commonly caused by staphylococcus aureus and late infections most commonly by staphylococcus epidermidis, although infections by staphylococcus lugudensis [3] streptococcus bovis, mitis and sanguis, pseudomonas [4], enterococci and fungi and even Mycobacterium fortuitum [5] have been described. Pacemaker infections present as local discharge, inflammation or abscess, erosion of part of the pacemaker system with secondary infection, or, more rarely, with endocarditis, sepsis and positive blood cultures. Pacemaker endocarditis refers to the presence of infective material in relation to the pacemaker system, in the veins leading up to the heart, or the chambers or the valves of the right heart. Pacemaker endocarditis is most commonly complicated by tricuspid valve vegetations [6], tricuspid regurgitation and occasionally by secondary pulmonary embolism [7]. Rare complications include SVC thrombosis [8] tricuspid stenosis [9] and paradoxical emboli [10].

Our patient presented 18 years subsequent to his first pacemaker implantation with bilateral pacemaker pocket and lead infection with staphylococcus epidermidis, complicated by tricuspid valve and right ventricular endocarditis and further complicated by SVC obstruction due to partly the sheer load of huge vegetations surrounding the 4 pacemaker leads in the SVC and partly by the stenotic changes in the vena cava as evidenced by the extensive backfilling of the azygous system on the contrast CT. The infection of the pacemaker pocket and the lead and the subsequent endocarditis could have been predicted on the basis of previous three reexplorations on the right side, one for slipped lead, the second for erosion and the last for unsuccessful percutaneous lead extraction, and one on the left side for elective pulse generator change.

There is general consensus that once there is pacemaker pocket or lead infection, removal of the whole pacemaker system followed by a course of appropriate antibiotics results in the best prospect for long term eradication of infection [11-14,7]. When pacemaker lead or pocket infection is complicated by vegetations on the leads, heart valves or chamber endocardium or when there is secondary pulmonary embolism, removal of the entire device is more urgently indicated. Pacemaker leads can be extracted percutaneously or after median sternotomy with cardiopulmonary bypass. Percutaneous extraction is preferably done under TOE guidance when there are no complicating factors like vegetations or endocarditis. Percutaneous lead extraction has been advanced by techniques initially described by Byrd [15,16] and perfected by other operators later [12,17,18]. The technique essentially involves counter traction to the myocardium by a plastic sheath passed over the lead while traction is applied to the lead tip by a stylet which passes to the tip of the lead through a central channel in it and which locks it by uncoiling it. Percutaneous extraction can fail, or may be complicated by non fatal pulmonary embolism [19], avulsion of tricuspid valve leaflet [18] or lead tip fracture with resulting vegetation [20]. TOE has been of value not only to diagnose the above complications, with the exception of PE, before the patient leaves the catheterization lab, but also to guide the mode of pacemaker lead removal. Generally lead removal by percutaneous traction in the presence of vegetations less than 1 cm is considered relatively safe, although one study described successful percutaneous traction in 9 out of 12 patients, with vegetations larger than 1 cm, with pacemaker endocarditis, with no post procedure secondary pulmonary embolism [21]. Authors of one study of 53 patients with pacemaker endocarditis, out of which 29 had transvenous extraction with locking stylets and sheaths, reported one surgical conversion, no tamponade and no major pulmonary embolism. They recommended that those patients who have vegetations that might obstruct main pulmonary artery should have removal under cardiopulmonary bypass [12].

As a general rule, however, in pacemaker endocarditis complicated by lead, tricuspid valve or RA or RV vegetations, removal of pacemaker leads is best done through median sternotomy, employing cardiopulmonary bypass and debriding the right heart chambers and the tricuspid valve, under direct vision, of the infected vegetations and debris. This offers the best method of achieving long term eradication of infection from the chambers of heart. Although removal of infected pacemaker system without employing cardiopulmonary bypass, under inflow occlusion, has been described [22], the authors would not advocate this procedure except under exceptional circumstances. However, the author (PK) has removed a stuck right ventricular lead, in a different patient, through a purse string in the right atrial appendage, without taking recourse to cardiopulmonary bypass, in the absence of lead, chamber or valve vegetations, and with TOE monitoring. There has been one report of removal of a pacemaker lead sticking out of right atrium without cardiopulmonary bypass by using a purse string around the projecting lead [23].

Pacemaker endocarditis only rarely involves the tricuspid valve to the extent of destroying it and mostly debridement of vegetations from the valve and preservation of the valve itself suffices. However, occasionally excision of the tricuspid valve with or without valve replacement [24], tricuspid valve reconstruction with PTFE chordae [25] and balloon dilatation 4 years after pacemaker endocarditic tricuspid stenosis [9] have been described. Taira reported tricuspid valve stenosis related to subvalvar adhesion of a non infected loop of an excessively long ventricular lead [26] and Hagers reported acquired tricuspid stenosis in a patient with recurrent pacemaker lead endocarditis, further complicated by a paradoxical septic embolism through a PFO [10]. Surgery for pacemaker endocarditis may be accompanied by additional procedures [14] apart from tricuspid valve surgery, including pulmonary embolectomy [27]. Hong et al described removal of 6 entrapped endocardial pacemaker leads with concomitant redo coronary artery bypass grafting [28].

Lead perforation during implantation is a recognised but rare complication of transvenous pacemaker implantation. It may present by a rising stimulation threshold, an RBBB pattern from an RV lead, intercostal muscle or diaphragmatic contraction, friction rub after implantation, pericarditis, pericardial effusion, cardiac tamponade or rarely, no symptoms [1].

It was only at median sternotomy after dissecting the large granulomatous mass found adherent to the under surface of the sternum near the manubrium that it was realised that there had been a perforation of the right atrium with one of the right atrial leads, the lead having exited the right atrial appendage, and then migrated a considerable distance to lie adherent to the under surface of the sternum. This could only have happened at the time of the previous unsuccessful attempt at percutaneous extraction of the pacemaker leads which had been complicated by cardiac arrest after which the extraction was abandoned and a new pacemaker system implanted from the left side. Since these leads were not connected to the pulse generator which had been removed, the lead would have got further exteriorised with cardiac contractions without at the same time giving rise to frank bleeding into pericardial cavity. Obliteration of pericardial cavity by adhesions at sternotomy confirms this presumed course of events.

Presence of huge vegetations along the entire tract of the leads including right atrial and right ventricular endocardium and the tricuspid valve as well as the entangling of the tricuspid valve chordae with the RV leads mandated excision and replacement of tricuspid valve with a prosthetic valve.

There has been a tremendous improvement in prosthetic valve thrombosis in tricuspid position since Thorburn reported 20% incidence in monoleaflet models [29]. Subsequent reports, corresponding to a mixture of newer valve models, showed incidence lower than 0.7% per patient year [30,31]. Presentation may include fatigue, syncope, distended neck veins, signs of fluid retention, muffled valve clicks and middiastolic or pansystolic murmurs in tricuspid area. Confirmation by TTE requires presence of the following criteria: 1) high transvalvular gradients – mean of 6 mm Hg or higher, and peak of 15 mm Hg or higher 2) transvalvular gradients 50% or higher than observed before 3) visible thrombus on the prosthetic valve 4) inability to demonstrate 2 different mobile echoes representing the valve leaflets in a high quality image. Inability of the valve leaflets to either fully open or close on fluoroscopy employing multiple acquisition angles including the side view, with the disks parallel to the beam is generally considered indicative of a stuck valve. The diagnostic criteria for thrombosis on TOE were limited leaflet motion and/or visible thrombus [32].

The low velocity of blood across the tricuspid valve prosthesis makes it especially prone to thrombosis. There are well documented conditions that predispose to thrombosis. Probably the commonest is a sub therapeutic INR, with the mechanical tricuspid prosthesis requiring an INR in the range of 3 to 4. A change to low molecular weight heparin in place of heparin prior to surgery is yet another common predisposing cause [33]. Hypercoagulable states like pregnancy [34], hypereosinophilic syndromes [35], malignancy [36], low flow states like Ebstein's anomaly [37] and use of Biomedicus Centrifugal Pump [38], drugs like Rifampicin, Dicloxacillin [39], HRT [40], Phenytoin [41], mechanical rather than bioprosthetic valves [42], all predispose to a greater likelihood of developing prosthetic valve thrombosis.

Our patient, who was continued on vancomycin and rifampicin postoperatively, required doses of warfarin in excess of 15 mg daily, in all probability due to the known facilitation of the induction of warfarin degradation by rifampicin. He presented with intermittent syncope and profound hypoxia responsive to increasing inspired fio_2_. TTE, TOE and fluoroscopy were clearly confirmatory. In addition, contrast CT scan demonstrated secondary pulmonary embolism and residual SVC obstruction. Clinical, radiological and echocardiographic resolution after Alteplase thrombolysis was instantaneous after one week of aggressive anticoagulation failed.

Although tricuspid valve thrombosis may occasionally respond to high intensity anticoagulation, thrombolysis should be the first line of treatment in these patients and is almost always successful. Failure of thrombolysis in symptomatic patients, particularly when both leaflets are stuck, is an indication of surgery [32] which shall usually involve re-replacement, and rarely, thrombectomy with or without rotation of the disk.

We believe this case report is unusual on account of the following: 1. The fact that a perforated right atrial lead lying more than an inch outside right atrial appendage was discovered 13 years after the attempted unsuccessful removal 2. Presentation of pacemaker endocarditis with a massive load of vegetations along the entire pacemaker lead tract in SVC, right atrial endocardium, tricuspid valve and right ventricular endocardium, leading to a functional and structural SVC obstruction 3. Requirement of an unusually large dose of warfarin postoperatively occasioned, in all probability, by antibiotic drug interactions 4. Presentation of tricuspid prosthetic valve thrombosis uniquely as vasovagal syncope and isolated hypoxia and near instantaneous resolution of tricuspid prosthetic valve thrombus with Alteplase thrombolysis.

## Competing interests

The authors declare that they have no competing interests.

## Authors' contributions

PK conceived, designed and drafted the manuscript and was the principal operating surgeon and consultant in charge of patient's care. KA and KJ assisted in the operation and collected various imaging data. WB was the primary cardiology consultant responsible for the care of the patient. All authors revised the manuscript and made important intellectual contributions.
